# Caudal regression syndrome and popliteal webbing in connection with maternal diabetes mellitus: a case report and literature review

**DOI:** 10.1186/1757-1626-1-407

**Published:** 2008-12-19

**Authors:** Ali Al Kaissi, Klaus Klaushofer, Franz Grill

**Affiliations:** 1 Ludwig Boltzmann Institute of Osteology, Hanusch Hospital of WGKK and AUVA Trauma Centre Meidling, 4th Medical Department, Hanusch Hospital, Vienna, Austria; 2 Orthopaedic Hospital of Speising, Paediatric Department, Vienna, Austria

## Abstract

**Introduction:**

Most cases of caudal regression are sporadic or associated with gestational/maternal diabetes. The condition is thought to be part of a spectrum including imperforate anus, sacral agenesis and sirenomelia. Infants of diabetic mothers have two to three times the average incidence of congenital anomalies.

**Case Presentation:**

We report on a 7-year-old boy from non-consanguineous family in Austria was born with features of caudal regression syndrome. A constellation of malformation complex such as caudal regression syndrome and anorectal malformation complex were evident at birth. Of great interest was the abnormal articulation between the pelvis and the remaining abnormal spine. Spinal-pelvic instability, dislocation of the hip, and knee-flexion contracture associated with popliteal webbing were the major orthopaedic abnormalities.

**Conclusion:**

We showed that an offspring of a diabetic mother was at significant risk of developing caudal regression syndrome. Our present patient demonstrated type1 of Welch and Aterman classification. There was total sacral agenesis associated with subtotal lumbar agenesis. The lowest vertebrae were resting above an iliac amphiathrosis. We strongly encourage primary care providers to discuss the consequences of maternal diabetes mellitus as part of routine anticipatory guidance for antenatal/prenatal management. Careful diabetic control in the preconceptional period and the first eight weeks of pregnancy may lower the chances of congenital anomalies.

## Introduction

Caudal regression syndrome and caudal dysgenesis syndrome are broad terms that refer to a heterogenous constellation of congenital caudal anomalies affecting the caudal spine and spinal cord, the hindgut, the urogenital system, and the lower limbs. About 15–25% of mothers of children with caudal regression syndrome have insulin-dependant diabetes mellitus [1]. Welch and Aterman [2] classified congenital sacral anomalies into 4 distinct clinical types. (1) A non-familial type associated with maternal diabetes mellitus showing complete absence of the sacrum and lower vertebrae with multiple congenital anomalies, (2) agenesis of the distal sacral or coccygeal segments, (3) hemisacral dysgenesis with presacral teratoma, and (4) hemisacral dysgenesis with anterior meningocele. Autosomal dominant inheritance was suggested for the last three types. Patients with caudal regression syndrome lack motor function below the level of the remaining normal spine, similar to those with myelomeningocele. In myelomeningocele, however, sensory nerve function is impaired below the level of the affected vertebrae. In caudal regression syndrome, sensation tends to be present at much more caudal levels. Infants of diabetic mothers have two to three times the average incidence of congenital anomalies.

## Case Presentation

**Figure 1:**
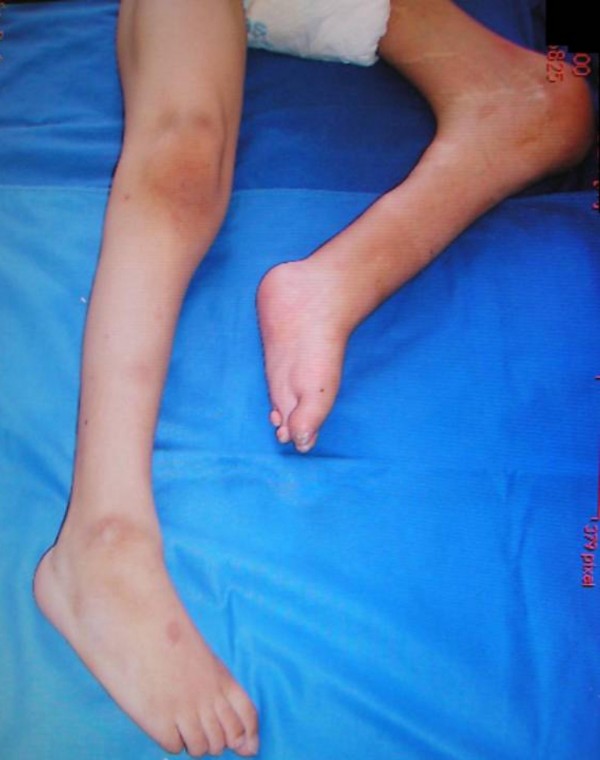
**Fixed flexion deformity of the knee overwhelmed by extensive popliteal webbing**.

**Figure 2:**
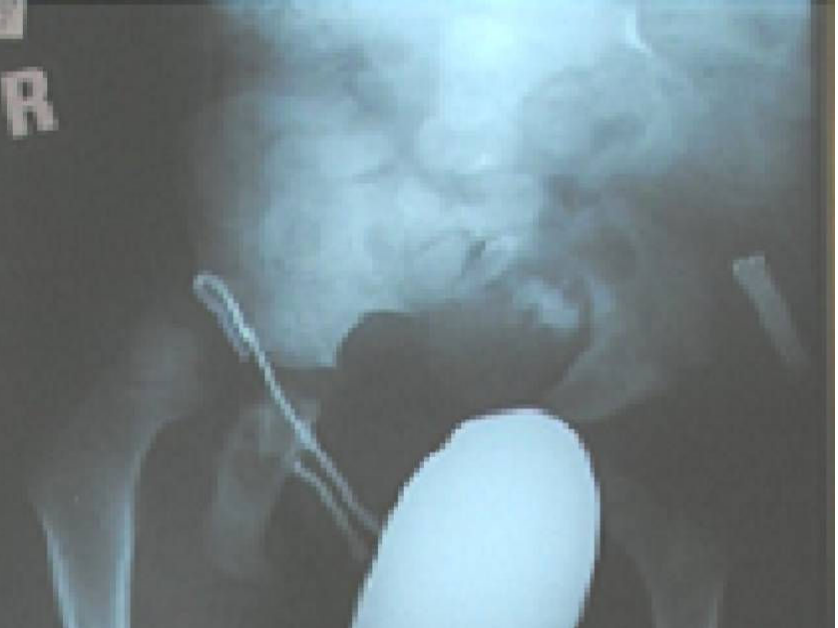
**Anteroposterior pelvis radiograph showed total agenesis of the sacrum with subtotal lumbar agenesis**. The lowest lumbar vertebrae were resting above an iliac amphiarthrosis.

**Figure 3:**
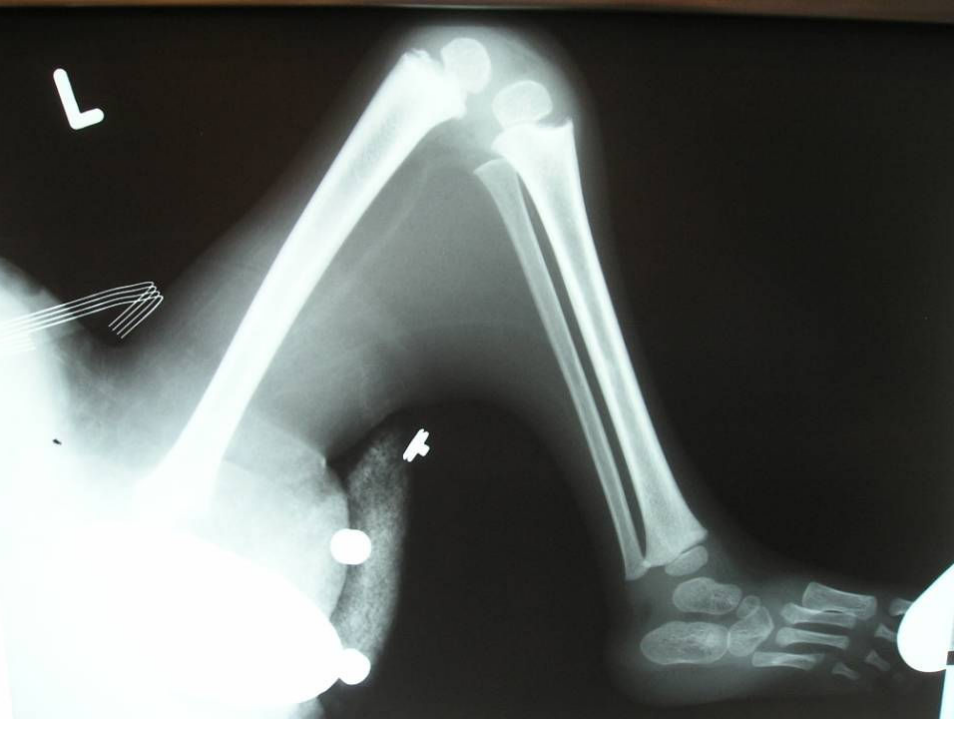
**Lateral lower limb radiograph showed fixed deformity of the left knee associated with soft tissue web behind the knee extends more than halfway down the tibia to the ankle associated with oligodactyly associated with significant dysplasia of the tarsal bones, agenesis and hypoplasia of the metatarsals and agenesis of the fourth metatarsophalangeal and proximal phalanx**.

The boy was referred to the orthopaedic department at the age of 3 years (fig 1). A loop colostomy in the left upper quadrant was performed at birth for his imperforate anus. He was the offspring of non-consanguineous parents. Family history revealed a non-insulin diabetic mother. There was no other history of significance. At birth he had lower limb anomaly and imperforate anus. There was a history of urine incontinence. Spinal-pelvic instability, dislocation of the hip, and knee-flexion contracture associated with popliteal webbing were the prominent orthopaedic abnormalities. Clinically he showed no dysmorphic craniofacial features and he was of normal intelligence. Hearing and vision were normal. No associated upper limb abnormalities. There was total sacral agenesis and partial lumbar spinal agenesis. His left hip was flexed and partially abducted because of a ptyrigium. Equinovarus deformity of the left foot was present as well. His motor development was normal over the right lower limb but paralyses and loss of sensations over the left lower limb was evident. He was able to move by means of his normal motor right lower limb. Radiographic documentation showed total agenesis of the sacrum with subtotal lumbar agenesis. The lowest lumbar vertebrae were resting above an iliac amphiathrosis (fig 2). Lateral lower limb radiograph showed fixed deformity of the left knee associated with soft tissue web behind the knee extends more than halfway down the tibia to the ankle associated with oligodactyly associated with significant dysplasia of the tarsal bones, agenesis and hypoplasia of the metatarsals and agenesis of the fourth metatarsophalangeal and proximal phalanx (fig 3). For his left hip ptyrigium, correction has been done by the plastic surgeon, followed by open reduction of the left hip, faced with the fixed flexion contracture of the left knee. Elected amputation (subtrochanteric amputation) and prosthetic fitting might be our choice. The advantage of subtrochanteric amputation over amputation at a more distal level is the ease of prosthetic fitting without having to deal with flexed femurs due to hip flexion contracture.

## Discussion

Caudal regression syndrome was first proposed by Duhamel [3], based on his observation that sirenoid monsters have a number of abnormalities in addition to the characteristic synmelia or fusion of the lower limbs. He further described an anchipod type of sirenoid monster in which the lower limbs are flexed and externally rotated but remain independent. Rumplessness, a condition similar to sacral agenesis, has been noted in animals, particularly chickens [4]. Duraiswami [5] demonstrated that insulin injected into chick embryos could cause rumplessness. In 1959, Blumel et al, [6] first called attention to the increased incidence of diabetes in mothers of affected children, they observed that children with sacral agenesis have been born to mothers who had abnormal glucose tolerance tests but were not taking insulin. Rusnak and Driscoll [7] reviewed the cases of 1150 infants born to diabetic mothers. Three had sacral agenesis, making the incidence 1 out of every 350 births to diabetic mothers. Although it is tempting to attribute sacral agenesis to alterations in carbohydrate metabolism induced by diabetes or exogenous insulin injections. Welch and Aterman [2] postulated that sacral agenesis resulted when an embryo with a genetic predisposition to the condition was exposed to some factor in the diabetic uterus. This unknown stimulus could be insulin, antibodies to insulin, or some other abnormality of carbohydrate metabolism. Several children have been described in which a sacral defect is inherited and there is no association with maternal diabetes. They further described three forms of familial sacral agenesis; familial hemisacrum type I (Cohn-Bay-Nielson syndrome), familial hemisacrum type II (Ashcraft syndrome), and familial partial sacral agenesis. A few instances of caudal regression have been reported in siblings [8]. Chromosomal studies have been normal [9], with few exceptions. A wide spectrum of spinal and extra-spinal abnormalities in connection with caudal regression syndrome has been reported. Bohring et al., [10] reported 15 infants with agenesis of the lower vertebral column overlapping with axial mesodermal dysplasia and infants seen where the mother has diabetes. Versiani et al., [11] report on 2 infants with absent lower limbs. Both mothers had gestational diabetes. Currarino syndrome [12], described a similar disorder caused by mutation in the HLXB9 gene on chromosome 7q36. Also called the ASP association, the A stands for anal stenosis, ectopia or an imperforate anus. The S stands for either a crescentic bony defect or malsegmentation of the sacrum and the P a presacral mass, either an anterior meningocele, or a teratoma or cyst. A part from caudal regression syndrome, there seems to be an increase incidence of other malformation complex such as visceral anomalies in connection with maternal diabetes. Slavotinek et al., [13] reported three babies with situs ambiguous born to mothers with insulin-dependent diabetes mellitus. One had situs inversus with a neural tube defect and the other two asplenia or polysplenia. Kuehl and Loffredo [14] present evidence suggesting that situs abnormalities are significantly more common in offspring of diabetic mothers. Lin et al., [15] note the association between abnormalities of situs, features of oculo-auriculo-vertebral complex, and maternal diabetes. Martinez-Frias [16] presented data suggesting that the incidence of transposition of the great vessels and transposition of viscera was significantly increased in the offspring of diabetic mothers.

## Conclusion

It has been known that diabetes-antedating pregnancy can have severe adverse effects on fetal and neonatal outcomes. As early as in the 1940s, it was recognized that women who developed diabetes years after pregnancy had experienced abnormally high fetal and neonatal mortality. The basic pathognomonic features in our current patient were total sacral and partial lumbar spine agenesis in connection with maternal diabetes mellitus. Prenatal diagnosis by ultrasound is possible at 22 weeks of gestation, seen as sudden interruption of the spine due to absence of vertebrae and a frog-like position of the lower limbs. In a recent case report, CRS was diagnosed antenatally after detection of a large nuchal translucency, although the diagnosis was not confirmed until 16 weeks [17]

## Abbreviations

CRS: Caudal regression syndrome; HLXB9 gene: homeobox gene.

## Consent

Written informed consent was obtained from the parents for the purpose of publication of the manuscript and figures of their child. A copy of the written consent is available for review by the editor-in-Chief of this journal.

## Competing interests

The authors declare that they have no competing interests.

## Authors' contributions

All of the authors were involved in the clinico-radiographic assessment and finalising the paper. All authors have red and approved the final version of the paper.
